# Supplementation with fish oil reduces αβ 42 burden and shifts αβ precursor protein processing toward non-amyloidogenic pathways in a rat model of hyperglycaemic Alzheimer’s disease

**DOI:** 10.1017/jns.2025.10036

**Published:** 2025-09-01

**Authors:** Nurina Titisari, Ahmad Fauzi, Intan Shameha Abdul Razak, Nurdiana Samsulrizal, Hafandi Ahmad

**Affiliations:** 1 Department of Veterinary Preclinical Sciences, Faculty of Veterinary Medicine, Universiti Putra Malaysia, Serdang, Selangor, Malaysia; 2 Department of Veterinary Physiology, Faculty of Veterinary Medicine, Universitas Brawijaya, East Java, Indonesia; 3 Department of Veterinary Clinical Pathology, Faculty of Veterinary Medicine, Universitas Brawijaya, East Java, Indonesia; 4 Faculty of Applied Sciences, Universiti Teknologi MARA, Shah Alam, Malaysia

**Keywords:** Alzheimer’s disease, Brain, Diabetes mellitus, Neurone, Omega-3, αβ, amyloid beta, APP, αβ precursor protein, LPS, lipopolysaccharides, STZ, streptozotocin

## Abstract

This study examines the influence of fish oil on brain amyloidogenesis in hyperglycaemic Alzheimer’s disease animal models, emphasising the potential of omega-3 fatty acids in fish oil to prevent the development of Alzheimer’s disease. Thirty males of Wistar rats were divided into five groups: 1) control rats (NS); 2) rats supplemented with 3 g/kg of fish oil (NS+FO3); 3) rats injected via intraperitoneal (i.p) with Streptozotocin-Lipopolysaccharide (STZ-LPS); 4) rats injected with STZ-LPS (i.p) and supplemented with 1 g/kg of fish oil (STZ-LPS+FO1), and 5) rats injected with STZ-LPS (i.p) and supplemented with 3 g/kg of fish oil (STZ-LPS+FO3). The cerebral brain was extracted for examination, and the αβ precursor protein (APP) level was measured using an immunoassay kit, while αβ 42 expression was evaluated using immunohistochemistry staining. Brain amyloidosis-related genes were quantified using real-time Polymerase Chain Reaction (PCR). The results revealed that fish oil supplementation significantly increased APP levels and reduced αβ 42 accumulations in STZ-LPS rats. Moreover, the Apolipoprotein E, ε4 isoform (ApoE-4) and Beta-site APP-cleaving enzyme 1 (Bace-1) genes were downregulated while the Low-density lipoprotein receptor-related protein 1 (Lrp-1) gene was upregulated in STZ-LPS rats treated with fish oil, thereby elucidating the impact of fish oil on diminishing αβ buildup in the brain. Therefore, this study contributes to a growing body of evidence supporting dietary interventions as adjunctive strategies for the prevention or delay of Alzheimer’s disease progression in metabolic dysfunction.

## Introduction

Amyloidogenesis and brain insulin resistance are the two primary mechanisms underlying hyperglycaemia and Alzheimer’s disease, along with mitochondrial dysfunction, neuroinflammation, and oxidative stress.^(1)^ Amyloidogenesis is the growth or development of amyloid beta (αβ) structures. The αβ formation process begins with a misfolded protein and progresses to forming prefibrillar aggregates, which eventually aggregate into insoluble fibrils known as αβ fibrils.^(2)^ These fibrils are resistant to degradation and can accumulate in various tissues and organs, leading to a number of degenerative disorders, including Alzheimer’s disease.^(3)^ The vast majority of αβ theories in Alzheimer’s disease studies postulated that excessive αβ production or a failure to remove this peptide from the body causes αβ deposition and accumulation, which is thought to be involved in the development of neurofibrillary tangles leading to neuronal loss.^(4,5)^

Moreover, it is widely known that αβ is a normal product of cellular metabolism generated from proteolytic cleavage of a larger glycoprotein known as αβ precursor protein (APP). Normally, APP is processed by enzymatic digestion α- and γ- secretase to form harmless peptide fragments in the non-amyloidogenic pathway, resulting in soluble αβ.^(6)^ This soluble αβ can be transported from the brain into the blood across the brain-blood barrier by low-density lipoprotein receptor-related protein 1 (Lrp-1) to prevent αβ accumulation in the brain.^(7)^ However, in the diabetic brain, APP is processed in the amyloidogenic pathway, which is cleaved by β- and γ- secretase, resulting in whole-length αβ peptides.^(8,9)^ There are various lengths of αβ peptides, but the most common forms in Alzheimer’s disease are αβ 40 and αβ 42.^(10)^ αβ 42 is particularly crucial because it is more prone to aggregation and is thought to be more toxic than αβ 40.^(11)^

Meanwhile, it has been reported that fish oil rich in omega-3 fatty acids may influence APP processing in mouse models and cell culture experiments.^(12,13)^ Another cell culture studies mentioned that omega-3 fatty acids administration prevented αβ formation by decreasing and modifying β- and γ- secretase activities and also encouraged the non-amyloidogenic pathway.^(14,15)^ In fact, αβ levels were reduced by more than 30% due to omega-3 fatty acids supplementation.^(16)^ Consistent with these findings, the animal model outcomes further indicated that fish oil reduces αβ 42 accumulation in five-familial-Alzheimer’s-disease–mutation (5xFAD) transgenic mice by modulating microglia number and behaviour.^(17)^ Another study demonstrated that fish oil supplementation promoted αβ clearance from the brain into the circulation in APP-transgenic mice by restoring Lrp-1 expression in the brain capillary endothelial cell.^(18)^ Hence, additional exploration was carried out toward fish oil rich in omega-3 fatty acids on APP processing and associated genes that may modulate brain amyloidogenesis.

## Methodology

### Experimental animals

Male Wistar rats aged 6–8 weeks old and with a body weight of 250 ± 20 g were used to generate a hyperglycaemia Alzheimer’s disease animal model using Streptozotocin-Lipopolysaccharide (STZ-LPS) induction following Murtishaw’s protocol with modification.^(19)^ Sample size (n = 6 per group) was determined on the resource-equation method outlined by Arifin & Zahiruddin (2017).^(20)^ The animals were block-randomly assigned to groups using a computer-generated sequence, stratified by baseline body-weight tertiles to ensure group balance. In this study, STZ and LPS were administered via intraperitoneal (i.p) injection, whereas in Murtishaw’s study, STZ was injected intracerebroventricularly, and LPS was given intraperitoneally. The animals were purchased from Anilab company (Bogor, Indonesia). Prior to the experiment, the animals were acclimated in the experimental animal laboratory of the Faculty of Medicine, Universitas Brawijaya, for a period of seven days. Throughout the experiment, all animals were housed as three rats per cage (polycarbonate, 465×300×185 mm) and had unlimited access to water and standard rat feed (Ratbio, Citra Ina Feedmill, Indonesia) ad libitum under well-ventilated conditions of 12 h of light/dark cycles; body weight, food, and water intake were recorded weekly. All procedures complied with ARRIVE 2.0 guidelines and were approved by the Institutional for Animal Care and Use Committee (IACUC), Universiti Putra Malaysia (UPM), Selangor, Malaysia (UPM/IACUC/AUP-R017/2022).

### Experimental protocol

Twelve rats were injected with normal saline (NS) as a control group; 1) Animals received NS injection (i.p) + NS (per oral (p.o)) (n = 6; NS group); 2) Animals received NS injection (i.p) + fish oil 3 g/kg (p.o) (n = 6; NS+FO group). Meanwhile, eighteen rats were injected with STZ to develop hyperglycaemia for three days. On day 7^th^ after the first injection of STZ, these animals were induced with LPS for Alzheimer’s disease development for another seven days and then divided into three groups; 3) Animals received STZ-LPS injection (i.p) + NS (p.o) (n = 6; STZ-LPS group); 4) Animals received STZ-LPS injection (i.p) + fish oil 1 g/kg (p.o) (n = 6; STZ-LPS+FO1 group); 5) Animals received STZ-LPS injection (i.p) + fish oil 3 g/kg (p.o) (n = 6; STZ-LPS+FO3 group). The oral administration of Menhaden fish oil (Sigma Aldrich, Cat no: F8020) was given using oral gavage at 8–9 am every day for 6 weeks. Thereafter, all rats were euthanized with an i.p injection of ketamine hydrochloride (100 mg/kg) combined with xylazine (10 mg/kg), and their brain tissue was collected for further examination.

### Experimental induction of hyperglycaemia-Alzheimer’s disease

Hyperglycaemia was induced in overnight fasted animals by intraperitoneal injection of 45 mg/kg STZ (Santa Cruz, cat no: SC-200719). The STZ was dissolved in sodium citrate buffer (0.01M, pH 4.5) on three successive days to generate hyperglycaemia conditions. After one week of injection with STZ, blood glucose was measured using a digital glucometer. Only rats with fasting blood glucose levels of 250 mg/dl and above were considered hyperglycaemic and included in the experiment. Next, only the successful hyperglycaemic rats were induced with 250 µg/kg LPS from E. coli O111:B4 (Sigma-Aldrich, cat no: L2630) once a day for seven successive days to create an Alzheimer’s disease animal model. The LPS was diluted in physiological saline (0.9% NaCl solution) and administered i.p.

### Preparation of brain protein extraction

Following the manufacturer’s instructions, the brain sample was extracted using a Pro-prep protein extraction solution (Intron Biotechnology, cat no: 17081). Approximately 10 mg of cortical and hippocampus tissues are collected using a scalpel. In 600 µl of PRO-PREP solution, the tissues are then homogenised using a mortar. Next, the microtube should be filled with 1.5 ml of solution and centrifuged at 13,000 × g for 10 min. The temperature of the chiller is set at 20°C, and the samples are left to incubate for 30 min. Afterward, a cold centrifuge was used to centrifuge the sample at a speed of 13,000 × g for a duration of 5 min. Finally, the supernatant was carefully transferred into a new 1.5 ml microtube and prepared for Enzyme-linked immunosorbent assay (ELISA).

### Enzyme-linked immunosorbent assay (ELISA) procedures

The concentration of APP was measured by ELISA kits (Bioassay Technology Laboratory, cat no: E1859Ra) according to the manufacturer’s instructions. Briefly, 50 µl of the standard solution was added to the standard well, while 40 µl of the brain sample and 10 µl APP antibody were added to the sample wells. Next, 50 µl of streptavidin-horseradish peroxidase was added to the standard and sample wells. The plate was homogenised and then covered with sealer before incubating at 37°C for 60 min. Thereafter, the sealer was removed, and the plate was washed five times with a wash buffer of roughly 0.35 ml/well for 30 s for each wash. The plate was dried by tapping it on the tissue paper, and then 50 µl of substrate solutions A and B were added to each well sequentially. Next, the plate should be incubated with the new sealer in the dark for 10 min at 37°C. Finally, 50 µl substrate stop solution should be added, and then it should be inserted into the microplate reader (Bio-Rad, USA) within 10 min after adding the stop solution. The absorbance was measured at a wavelength of 450 nanometres (nm).

### Immunohistofluorescence analysis

Paraffin-embedded right-hemisphere tissue was cut at 4 µm in the sagittal plane, mounted on poly-L-lysine slides, deparaffinised, re-hydrated, permeabilised for 5 min in 0.2% Phosphate-Buffered Saline (PBS) triton X-100, and blocked with 1% Bovine Serum Albumin (BSA) (30 min; at room temperature). Sections were incubated overnight at 4°C with primary antibodies against αβ 42 (Bioss; cat no: bsm-0107M; 1: 100). After three washes with PBS, slides received goat anti-rabbit immunoglobulin G tetramethylrhodamine isothiocyanate (IgG-TRITC) (Thermo; cat no: A-11077; 1: 1000; 30 min; at room temperature) and 4′, 6-Diamidino-2-Phenylindole (DAPI) (Invitrogen, cat no: D1306; 1: 1000; 5 min). The slides should then be washed three times with PBS, mounted, and covered with glass. Images were acquired on an Olympus FV1000 fluorescence microscope, and the immunofluorescence staining quantification was processed using ImageJ (version 1.52q, USA) image software. Firstly, a blinded observer defines the region of interest (ROI) of the cortex in each slide at a magnification of 200× with a full pixel count of 800×600. Next, the ImageJ ‘Measure’ command was used to obtain the integrated-density value for the red channel. Data were expressed as the percentage of the ROI area that was positively stained by αβ 42 immunoreactivity. Five sections per animal were analysed, and the resulting percentages were averaged to yield a single value per rat.

### Ribonucleic acid (RNA) isolation and complementary deoxyribonucleic acid (cDNA) synthesis

The initial process in obtaining the cDNA template was to extract total RNA from brain tissue using the total RNA mini kit solution (Geneaid biotechnology company, cat no: RT100) according to the manufacturer’s instructions The concentration and purity of RNA were subsequently determined using NanoDrop™ (Thermo Fisher Scientific, USA) by measuring absorbance at 260 and 280 nm. The isolated total RNA was then stored in a freezer –80°C until use.

The cDNA was synthesised from the RNA sample in the second process using ReverTra Ace™ PCR RT Master Mix (Toyobo company, cat no: RT100) according to the manufacturer’s protocol. To begin, the genomic DNA was removed from the sample using DN master mix (with gDNA remover), and the sample was incubated at 37°C for 5 min. Next, the total RNA sample was reverse transcribed by adding RT Master Mix II and the sample was incubated at 37°C for 15 min to activate the reverse transcriptase enzyme. The RNA sample was then heated for 15 min at 95°C using conventional PCR (Biorad, USA). Finally, the cDNA template was frozen at –20°C for further use.

### Real time-quantitative polymerase chain reaction (RT-qPCR) procedures

The specific primer pairs for six Alzheimer’s disease-related genes and one reference gene using the Integrated DNA technologies (IDT) programme (Table 1). Gene sequences were obtained from the Gene Bank^®^ database for rats. BLAST searches were performed to confirm the gene specificity of the primer sequences, and the results showed an absence of multi-locus matching at individual primer sites. The β-actin gene was used as an internal control or housekeeping gene to determine the quantity and quality of cDNA, which was subsequently employed as a standard to estimate gene expression.

**Table 1.** Primers for brain amyloidogenesis-related genes

Gene symbol	Gene name	Primer Forward (F)/ Reverse (R)	Genbank	Amplicon size (bp)
ApoE-4	Apolipoprotein E ε4	CAATGATCATCCCTCACCTACG TATTAAGCAAG GCCACCG	NM_001270683.1	101
Lrp-1	Low-density lipoprotein receptor-related protein 1	ATC GCA GGC AAC ATC TAC TG CTT GTC TAG ACC CTG GGA AAT G	NM_001130490.1	105
Bace-1	Beta-site APP-cleaving enzyme 1	CACTCTGCCTCATGGTATT CCCACTGGCCTCCTTATTT	NM_019204.2	105
ACTB	β-actin	CCTAAGGCCAACCGTGAAA CAGAGGCATACAGGGACC	KJ696744.1	103

Each sample was then run in two technical replicates, and their results were documented as cycle threshold (Ct) values. The Ct values of the genes were averaged, and their relative expression levels to the housekeeping genes were calculated using the comparative ΔCt method using the following equation: ΔCt = number of target protein genes – housekeeping gene. Meanwhile, fold change (ΔΔCt) to measure gene expression was calculated using this equation: ΔΔCt = ΔCt (sample) – ΔCt (sample reference). The comparative ΔCt approach using β-actin as the reference gene and the NS group as the calibrator (NS = 1). Fold-changes for all other groups were expressed relative to this baseline.

### Statistical analysis

Statistical analyses were undertaken using SPSS (version 20.0; SPSS Inc.). All results, except for feed and water intake, are expressed as mean ± standard error of the mean (SEM). The one-way ANOVA followed by a post hoc Tukey test was used to compare among groups. Differences were regarded as significant when P < 0.05.

## Result

### Body weight, feed, and water intake

Table 2 summarises the means and SEM for the animal model’s average body weight. The groups of rats receiving NS induction (NS and NS+FO3) exhibited a consistent weekly rise in body weight, while the groups treated with STZ-LPS induction (STZ-LPS, STZ-LPS+FO1, STZ-LPS+FO3) had an ongoing and progressive weight loss beginning in week 3. In comparison to the NS group (261.00 ± 6.21), the STZ-LPS group exhibited a reduced body weight starting in week 3 (225.83±10.58; P < 0.05). The STZ-LPS FO1 group demonstrated a significantly lower body weight starting in week 4 (222.50 ± 3.78; P < 0.05), while the STZ-LPS FO3 group showed a significant difference from week 6 (232.00 ± 22.87; P < 0.05), compared to the NS group (286.67 ± 15.93 and 319.00 ± 16.34, respectively).

**Table 2.** Effect of fish oil supplementation on body weight (g) in STZ-LPS-induced rats

Groups	Week 1	Week 2	Week 3	Week 4	Week 5	Week 6
NS	255.33 ± 6.89^a^	253.17 ± 7.29^a^	261.00 ± 6.21^b^	286.67 ± 15.93^b^	294.00 ± 15.65^b^	319.00 ± 16.34^c^
NS+FO3	248.00 ± 8.57^a^	250.50 ± 9.87^a^	259.67 ± 9.29^b^	279.83 ± 9.24^b^	298.83 ± 8.99^b^	307.33 ± 10.23^bc^
STZ-LPS	243.67 ± 4.28^a^	243.33 ± 6.95^a^	225.83 ± 10.58^a^	211.67 ± 11.63^a^	202.67 ± 13.04^a^	203.83 ± 11.85^a^
STZ-LPS+FO1	249.50 ± 4.69^a^	247.67 ± 4.87^a^	246.67 ± 5.33^ab^	222.50 ± 3.78^a^	225.50 ± 3.96^a^	213.67 ± 7.66^a^
STZ-LPS+FO3	251.00 ± 5.15^a^	252.83 ± 5.15^a^	246.17 ± 8.22^ab^	238.00 ± 15.38^ab^	242.00 ± 19.73^ab^	232.00 ± 22.87^ab^

Values are mean ± SEM for six rats in each group. Values with different superscripts ^a,b,c^ in a column differed significantly at P < 0.05 due to time effects.

**Figure 1.**
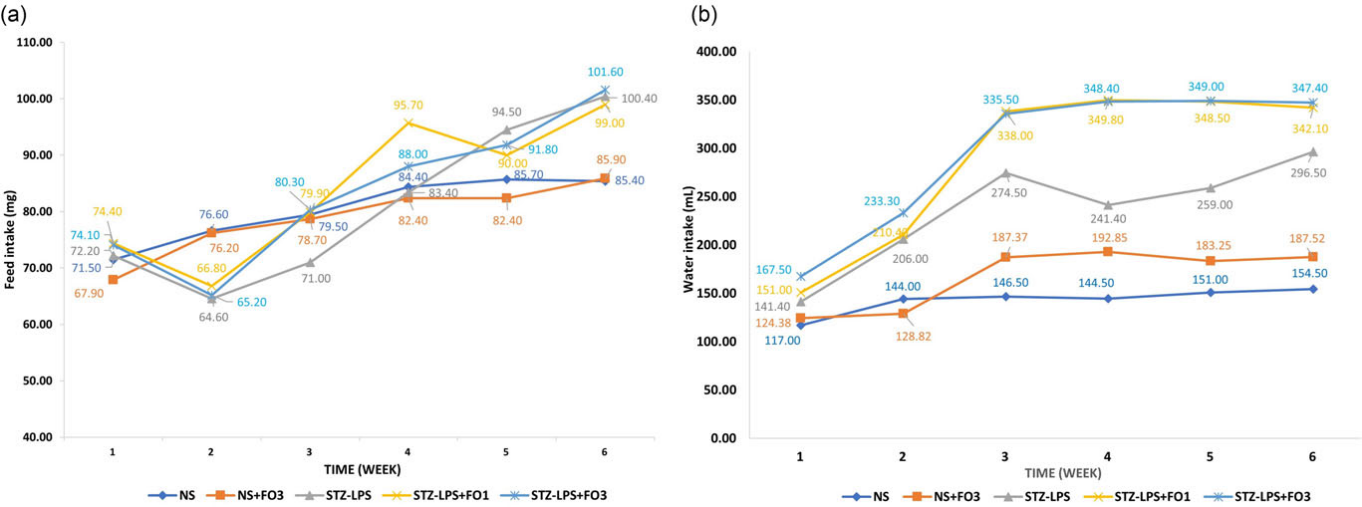
Mean daily feed consumption (left image) and mean daily water consumption (right image) were recorded for each cage (three rats per cage, n = 2 cages per group) over the 6-week study.

Feed and water intake were recorded for each cage, with each cage housing three rats according to their respective groups (Figure 1). Feed intake in the NS and NS+FO3 groups showed a steady increase throughout the 6-week study. In contrast, the STZ-LPS-induced groups (STZ-LPS, STZ-LPS+FO1, and STZ-LPS+FO3) exhibited a decline in feed intake during week 2 but subsequently surpassed the NS group by the end of the study. Daily water intake in the NS group remained relatively stable over 6 weeks, increasing from an average of 117 ml/d in week 1 to 155 ml/d in week 6 per cage. In comparison, the STZ-LPS group showed a marked increase in water intake, rising from 141 ml/d to 297 ml/d over the same period — an increase of approximately 110%. Fish oil supplementation in normal rats (NS+FO3) resulted in slightly higher water intake compared to the NS group. Meanwhile, STZ-LPS-induced rats receiving fish oil (STZ-LPS+FO1 and STZ-LPS+FO3) demonstrated higher water consumption than all other groups.

### Total brain αβ precursor protein (APP)

The means and SEM for APP levels were tabulated in Table 3. The STZ-LPS group showed the lowest APP concentration in the cortex (93.05 ± 15.04; P < 0.001) and hippocampus (133.63 ± 20.18; P < 0.05), compared with the NS group (489.48 ± 74.51 and 716.51 ± 82.26, respectively). In comparison to the STZ-LPS group, the level of APP in the cortex and hippocampus of STZ-LPS-induced rats increased significantly with the administration of fish oil at 1 g/kg (cortex 332.46 ± 43.51 and hippocampus 1427.82 ± 139.10; P < 0.001) and 3 g/kg (cortex 333.46 ± 17.28 and hippocampus 832.84 ± 242.49; P < 0.05). Meanwhile, the administration of 1 g/kg fish oil significantly increased (P < 0.05) APP levels in the hippocampus of STZ-LPS-induced rats in comparison to the NS group.

**Table 3.** Quantification of APP levels in the cortex and hippocampus of the rat brain by the ELISA method

Groups	APP (ng/ml)	
	Cortex	Hippocampus
NS	486.48 ± 74.51^###^	716.52 ± 82.25^#^
NS+FO3	495.82 ± 86.51^###^	764.17 ± 107.23^#^
STZ-LPS	93.05 ± 15.04***	133.63 ± 20.18*
STZ-LPS+FO1	332.46 ± 43.51^#^	1427.82 ± 139.10*/^###^
STZ-LPS+FO3	348.85 ± 35.68^#^	774.99 ± 95.72^#^

Data are presented as mean ± SEM (n = 6). *P < 0.05 compared to the NS group; ***P < 0.001 compared to the NS group. ^#^P < 0.05 compared to the STZ-LPS group; ^###^P < 0.001 compared to the STZ-LPS group.

### αβ 42 fluorescence intensity

**Figure 2.**
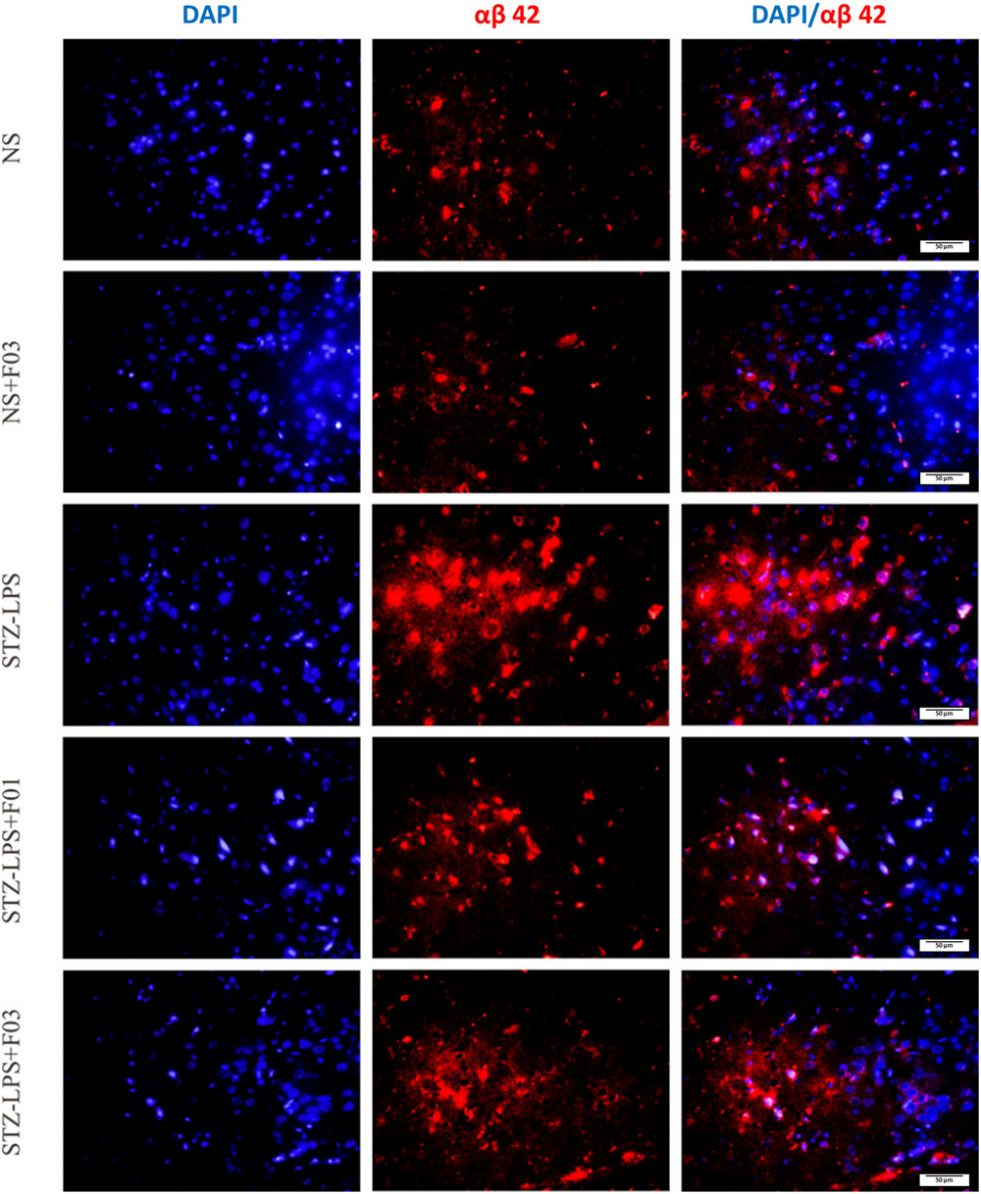
Representative immunofluorescence of αβ 42 in rat brain cortex. Sections were double-labelled with DAPI (blue signal) and αβ 42 (red signal). Images show, from left to right, the DAPI channel, the αβ 42 channel, and the merged view. Photomicrographs were captured with a 200 × total magnification using identical exposure settings for all groups; scale bar = 50 µm. Quantitative values reported in the text correspond to αβ 42-positive area ÷ total ROI area × 100 % (ROI = 800 × 600 pixels), averaged from five sections per animal.

**Figure 3.**
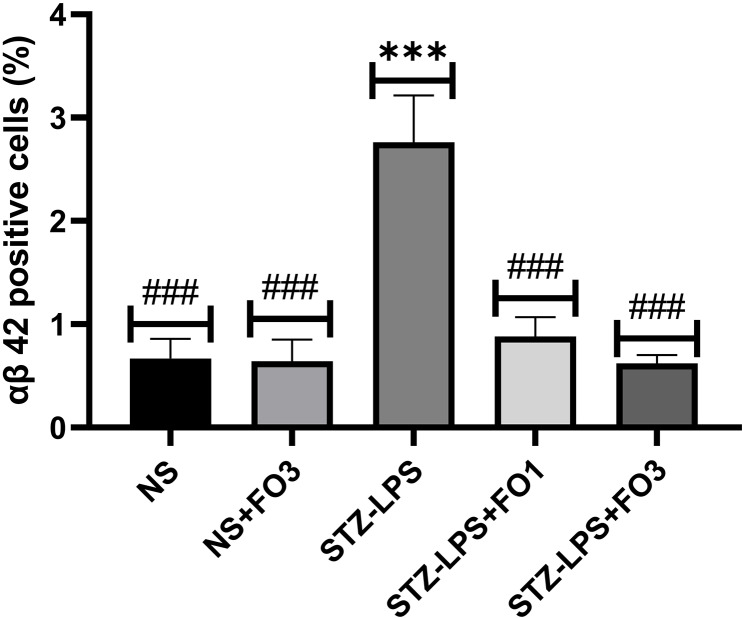
The fluorescence intensities were measured as a percentage of αβ 42 positive cells. All histological qualitative data are presented as means and standard errors of the means (SEM) (n = 6). ***P < 0.001 compared to the NS group; ^###^P < 0.001 compared to the STZ-LPS group.

The photomicrograph images of αβ 42 expression in the cerebral cortex were obtained using immunofluorescence staining (Figure 2). There appeared to be more αβ 42 immunoreactivity in the cerebral cortex in STZ-LPS-induced rats, and the quantification results showed that the STZ-LPS group had a significantly higher number of αβ 42 positive cells (2.73 ± 0.37; P < 0.001) than the NS group (0.68 ± 0.15) (Figure 3). In contrast, fish oil treatment at 1 g/kg (0.87 ± 0.15) or 3 g/kg (0.63 ± 0.07) in STZ-LPS-induced rats resulted in a dramatically lower percentage of αβ 42 (P < 0.001vs STZ-LPS group), confirming the visual differences shown in Figure 2.

### Relative expression analysis of αβ-related genes

**Figure 4.**
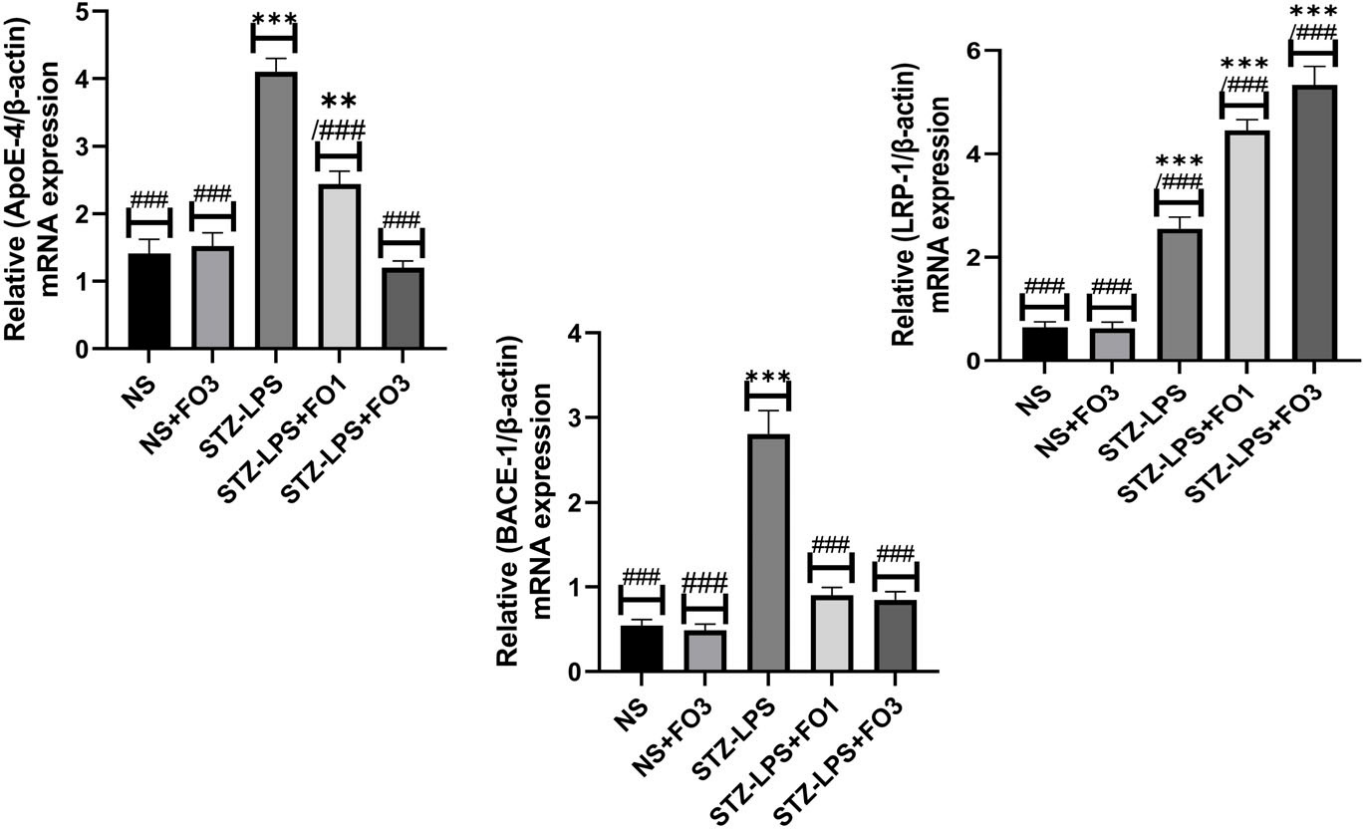
Effect of fish oil supplementation on the relative fold change of mRNA expression of all genes among groups. Values are shown as mean±SEM for 6 rats in each group. **P < 0.01 compared to the NS group; ***P < 0.001 compared to the NS group. ^###^P < 0.001 compared to the STZ-LPS group.

The fold change in mRNA expression of amyloidogenesis pathway-related genes in the STZ-LPS-induced group compared with the NS group (Figure 4). The RT-qPCR showed rats induced with STZ-LPS significantly increased (P < 0.001) fold changes in ApoE-4 (4.10 ± 0.20) and Bace-1 (2.81 ± 0.27) genes compared to the NS group (1.41 ± 0.22 and 0.54 ± 0.07, respectively). On the other hand, 1 g/kg of fish oil treatment in STZ-LPS-induced rats had lower (P < 0.001) fold changes in the ApoE-4 (2.44 ± 0.19) and Bace-1 (0.90 ± 0.09) genes compared to the STZ-LPS group. Meanwhile, the Lrp-1 gene fold changes were significantly higher (P < 0.001) in the STZ-LPS+FO3 group (5.33 ± 0.36) compared to the STZ-LPS group (2.55 ± 0.22), with markedly lower (P < 0.001 vs STZ-LPS group) fold changes of ApoE-4 (1.20 ± 0.10) and Bace-1 (0.85 ± 0.10).

## Discussion

The current study confirmed that STZ-LPS induction produced progressive weight loss from week 3 onward, and that the higher fish-oil dose moderated, but did not completely prevent, this decline. Physiologically, the body uses glucose from food to be converted into energy, but in diabetic conditions, glucose in the blood cannot enter the cells, causing hyperglycaemia.^(21)^ Consequently, the body breaks down body muscle and fat for energy, causing body weight loss. This study showed that fish oil supplementation in STZ-LPS-induced rats failed to mitigate the characteristic signs of diabetes in animal models, such as polydipsia, polyphagia, and weight reduction. Insulin deficiency results in elevated blood glucose levels, prompting the kidneys to excrete more urine to remove glucose, which in turn induces polydipsia and heightened water consumption.^(22)^

The current study reported that APP levels in the cortex and hippocampus were lower in the STZ-LPS group when compared to the other groups. This finding is in contradiction with in vitro studies that showed high glucose manipulation would increase APP protein levels in human neuroblastoma SH-SY5Y cells^(23)^ and also in human umbilical vein endothelial cells (HUVECs).^(24)^ On the contrary, in transgenic animal study revealed that diabetes increased but not full-length APP in STZ-induced APP transgenic mice brains.^(25)^ A similar study also reported that there were no changes in APP mRNA of APP/PS1 transgenic mice injected with STZ.^(26)^ It is demonstrated that increasing APP expression due to high glucose administration was not related to APP gene transcription.^(23)^ This indicates that hyperglycaemia does not affect APP levels in animal models, particularly in transgenic animals. However, in this study, STZ and LPS injections were used to generate a non-transgenic animal model for Alzheimer’s disease related to hyperglycaemia. Thus, our findings of lower levels of APP in STZ-LPS-induced rats require further investigation, especially on APP-related genes, to clarify the effect of glycaemia on APP expression.

Another important finding in this study is that fish oil supplementation increased APP levels in STZ-LPS-induced rats. APP is a type I single-pass transmembrane protein expressed in many cell types, including neurones. A study mentioned that overexpression of APP showed positive effects on cell health and growth.^(27)^ In fact, APP is essential for neurone generation, neurone plasticity, and neuroprotection.^(28)^ Transgenic mouse models study reported that lower APP is associated with poor cognitive performance, underlying the APP role in cognitive functions.^(29)^ Contrasting, it is widely accepted that excessive APP is known to be able to cause Alzheimer’s disease. Higher APP transcription was seen in the Alzheimer’s disease human brain compared to the control.^(30,31)^ Indeed, studies have reported that elevated APP expression is implicated in the pathogenesis of both early-onset and late-onset Alzheimer’s disease through duplication^(32,33)^ or gene mutation.^(34,35)^

Furthermore, a study also stated that an increase in APP would be accompanied by an increase in protease activity.^(23)^ On the other hand, it is generally acknowledged that APP can be processed through either an amylogenic or non-amylogenic pathway. When APP is processed in a non-amylogenic pathway, it will cleavage by α-secretase, resulting in an extracellular fragment called soluble APP alpha (sAPP-α) that has neuroprotective and neurotrophic functions and also the C-terminal fragment (CTF) of 83 amino acids (C83).^(36)^ C83 is then cleaved by γ-secretase, resulting in a non-amyloidogenic 3 kDa peptide (p3) fragment into the extracellular space and intracellular APP fragment known as APP intracellular C-terminal domain (AICD).^(37)^ Meanwhile, if the APP is processed through the amylogenic pathway, it produces αβ peptide, which builds up in αβ plaques.^(38)^

According to the RT-qPCR result in this study, the STZ-LPS-induced rats with fish oil treatment had lower fold changes in the Bace-1 and ApoE-4 genes and higher fold changes in the Lrp-1 gene. The Bace-1 gene expression is well known to play an important role in regulating APP processing pathways to undergo the amyloidogenic pathway.^(39)^ This study showed that fish oil administration could downregulate the Bace-1 gene activity, indicating that β-secretase is not produced, resulting in lower production of αβ, as in line with previous studies.^(13)^ Indeed, it is reported that omega-3 fatty acids, mostly contained in fish oil, will alter the APP processing to undergo non-amyloidogenic pathways.^(15)^ Furthermore, it is well established that the Bace-1 gene encodes the Bace-1 enzyme, a type 1 membrane protease that provides the proper topological orientation for APP cleavage at the β-secretase site, releasing a sAPP-β fragment and αβ precursor CTF of 99 amino acids (C99).^(40)^ Afterward, the CT99 is cleaved by γ-secretase, resulting in several forms of αβ peptides, such as αβ 40 and αβ 42, into the extracellular space and also releasing the APP intracellular domain (AICD) into the intracellular space.^(41)^

Moreover, αβ peptides, such as αβ 40 and αβ 42, which are associated with Alzheimer’s disease, are eliminated from the brain by the action of the Lrp-1 protein.^(42)^ This study found that fish oil supplementation increased the Lrp-1 gene expression in STZ-LPS-induced rats, demonstrating that the Lrp-1 protein was well generated. In line with this result, a study reported that fish oil increased the expression level of Lrp-1 and encouraged αβ clearance from the brain into the bloodstream.^(18)^ Additionally, the αβ peptides are then broken down by macrophages and hepatocytes or eliminated through the kidney or liver.^(43)^

The ApoE-4 gene expression in STZ-LPS-induced rats in this study was inhibited by fish oil treatments. Indicating that fish oil can lessen the elevated risk of Alzheimer’s disease in rats induced by STZ-LPS. Indeed, ApoE-4 has been connected to an increased deposition and decreased clearance of αβ, which play a role in the pathogenesis of Alzheimer’s disease.^(44)^ These results differ from research conducted by Lim and colleagues using aging Alzheimer’s disease transgenic mice that showed ApoE expressions was unchanged with the diet supplemented with 0.6% DHA.^(45)^ Moreover, another study revealed that ApoE is released and increased from activated glial cells in an in vivo brain injury model, which explains the close relationship between ApoE and neurodegenerative features.^(46)^ It was demonstrated that the internalisation of the released protein results in a concurrent increase in intracellular ApoE in degenerating neurones.^(47)^ In fact, two distinct mechanisms underlying this neuronal dysfunction are ApoE-4 accelerates αβ deposition in lipid rafts rich in cholesterol, and ApoE-4 modifies ApoE receptor signalling to reduce the protective effect against αβ accumulation.^(48)^

**Figure 5.**
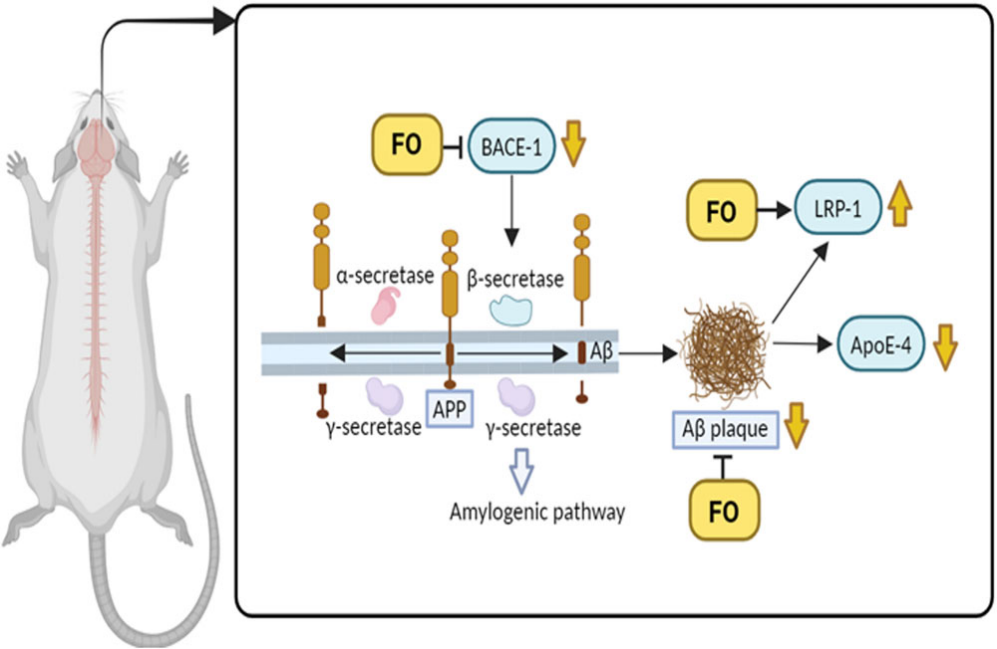
Brain rats with Alzheimer’s disease-related hyperglycaemia demonstrated the fish oil supplementation ability to prevent αβ plaque by encouraging APP to be processed in a non-amyloidogenic pathway and alter brain amyloidosis-related genes (created in BioRender.com).

Finally, based on the ELISA and RT-qPCR results, it is conceivable that the increasing APP levels due to fish oil supplementation were degraded through a non-amyloidogenic pathway, causing less accumulation of αβ in the extracellular brain (Figure 5). This is supported by immunofluorescence findings, which showed a smaller number of illuminated αβ 42 deposits in the cortex of rats induced by STZ-LPS. Although αβ 42 is less abundant in the brain than αβ 40, it is thought to be more pathogenic due to its tendency to clump and assemble into toxic plaques.^(49)^ In this study, there are not found any form of ‘Classical’ αβ plaques, which are dense-cored aggregated αβ with less dense halo or corona.^(50,51)^ The immunofluorescence images showed an accumulation of αβ 42 resembling the type of diffuse plaques, which are widely spread or scattered, amorphous deposits of αβ protein without a well-defined core. Even though Fan Liu and colleagues proposed that classical or focal plaques have greater potential as biomarkers for the neuropathological evaluation of Alzheimer’s disease than diffuse plaques^(52)^, however, another study discovered that diffuse plaques can still contribute to neurotoxicity.^(53)^ In fact, it is generally acknowledged that diffuse plaques are the first to appear, with classical or focal plaques emerging later in the Alzheimer’s disease pathologic process.^(54)^ Meanwhile, in parallel with previous studies,^(13,17)^ this study has demonstrated that fish oil administration can prevent the development of αβ plaques by lowering the buildup of amyloidogenic αβ. Indeed, the positive effect of fish oil on amyloidosis in cell studies or Alzheimer’s disease animal studies has been demonstrated by numerous scientific studies.^(45,55–57)^

Furthermore, the fish-oil doses employed here (1–3 g/kg per d, equivalent to 11–33 g/d for a 70-kg human adult via allometric scaling.^(58)^ Although higher than usual supplementation, comparable pharmacological intakes have been well-tolerated in humans — for example, 3.4 g of EPA+DHA (equivalent to ± 11 g of fish oil) for six months in adolescents^(59)^ and >2 g/d omega-3 fatty acids improving early outcomes in major depressive disorder.^(60)^ Clinical data also show no excess bleeding risk even when high omega-3 doses are combined with antiplatelet agents.^(61)^ Nonetheless, because current Food and Drug Administration guidance advises not exceeding 2 g of EPA+DHA/d (equivalent to ± 6.7 g of fish oil), future studies will down-titrate to clinically relevant doses to refine translational applicability.

This proof-of-concept study was conducted exclusively in adult male Wistar rats, as male pancreatic β-cells are markedly more sensitive to streptozotocin than female pancreatic β-cells. Therefore by using a male animal model allowed us to achieve stable hyperglycaemia without escalating the toxin dose and its off-target toxicity. Nevertheless, sex is a biological variable that can influence both metabolism and neurodegeneration, so future work must replicate these findings in females and explore sex-specific responses. In addition, because the fish-oil doses employed here are higher than typical human intakes, subsequent studies should test lower, clinically relevant doses, incorporate a greater number of animal models, and potentially include behavioural and cognitive assessments. Taken together, these factors indicate that our results provide potential, rather than definitive, evidence for the therapeutic utility of dietary omega-3 fattu acids in hyperglycaemia-associated Alzheimer disease-like pathology.

## Conclusion

Fish-oil supplementation reduced cortical αβ 42 deposition and shifted APP processing towards the non-amyloidogenic pathway in STZ-LPS rats, results that were associated with the down-regulation of Bace-1 and ApoE-4 and the up-regulation of Lrp-1. These molecular alterations suggest diminished synthesis and increased outflow of αβ, supporting a protective role of omega-3 fatty acids under diabetic conditions. Nevertheless, because the study used pharmacological doses, a singular sex, and a limited sample size, the results should be regarded as pre-clinical evidence of feasibility rather than definitive proof of clinical efficacy. Future investigations should incorporate female cohorts, employ human-equivalent dosing, increase the number of animal models, and extend to cognitive outcomes to establish the translational relevance of omega-3 fatty acids for Alzheimer disease-like pathology in diabetes.
